# Population Access to Hospital Emergency Departments: The Spatial Analysis in Public Health Research

**DOI:** 10.3390/ijerph19031437

**Published:** 2022-01-27

**Authors:** Wojciech Kisiała, Izabela Rącka, Katarzyna Suszyńska

**Affiliations:** 1 Department of Econometrics, Institute of Informatics and Quantitative Economics, Poznań University of Economics and Business, 61-875 Poznań, Poland; wojciech.kisiala@ue.poznan.pl; 2 Department of Management, Faculty of Social Sciences, Calisia University—Kalisz, 62-800 Kalisz, Poland; 3 Department of Investment and Real Estate, Institute of Management, Poznań University of Economics and Business, 61-875 Poznań, Poland; katarzyna.suszynska@ue.poznan.pl

**Keywords:** access to health care, health care services, hospital emergency departments, spatial analysis, public health management

## Abstract

The emergency medical services support the primary health care system. Hospital emergency departments (HEDs), which provide medical assistance to all patients in a state of emergency are of considerable importance to the system. When studying access to HEDs, attention should be focused on spatial relations resulting from the location of HEDs and the places of residence of the potential patients. The aim of the paper is to explain the level of spatial accessibility of HEDs and its changes as a result of organizational and spatial transformations of HEDs’ networks in Poland. The research was conducted within two time series, comparing the changes in the distribution of HEDs in 2011 and 2021. GIS techniques were used to measure the distances between emergency departments and places of residence. It was observed that the transformation of the spatial organization of the hospital emergency department network in 2011–2021 resulted in the overall improvement of the spatial accessibility of these facilities, reducing the distance between them and places of residence.

## 1. Introduction

One of the most basic social needs of human beings is security, satisfied by, e.g., properly designed health protection. Due to the modern lifestyles people lead, the risk of numerous civilization and social diseases, including those caused by various types of injuries, increases every year. Therefore, it is necessary to shape the health care system in such a way that it would give citizens a sense of security, especially with regard to the pathogenic effects of an accelerating pace of life in the modern world [1,2]. The importance of this type of activities is indicated and proved by their presence in the third goal of the sustainable development programme—2030 Agenda for Sustainable Development [3]. Since a person’s life and health in many cases is determined by the speed of medical actions taken, the problem of spatial organization of emergency medical services is of major importance [4,5,6,7].

Until the end of the 1990s, emergency medicine in Poland was obsolete, and emergency care was in fact limited to visits of the doctors and medical transport to hospitals. The emergency rescue system operating in Poland since the post-war years complemented the inefficient primary health care system, without guaranteeing comprehensive, adequate to the needs and sufficiently quick medical service in the case of sudden health or life-threatening events. The need for reforms in the field of emergency medical services, in addition to widespread social discontent, was also reflected in the mortality rate at the scene of accidents or deaths from sudden illnesses on the spot or on the way to the hospital, which significantly exceeded the international standards [8,9,10].

The increasingly visible imperfections in the functioning of the Polish emergency medical services and the positive effects of implementing modern medical systems in western European countries were incentives to organize similar structures in Poland. The works undertaken at the turn of the 20th and 21st centuries led to the implementation of the Act of 8 September 2006 on the Emergency Medical Services (EMS). According to its regulations, planning and organization of the system takes place mainly at the regional level (voivodeships), and the authorities responsible for the performance of the system’s tasks are voivodes (representatives of state government administration in regions). Supervision over the system is exercised by the Minister of Health.

The task of the voivodes is to prepare regional plans for the operation of the system, which defines, e.g., the number, location and method of coordinating the activities of the units. The act distinguishes between two types of units operating within the system: hospital emergency departments (HED) and emergency medical teams.

An important element of the introduced reforms was the creation of a hospital base of emergency medical services. Hospital emergency departments (HEDs), as organizational units of hospitals specializing in emergency care, are the place of first contact for people in a state of emergency. The main task of HEDs is to admit, perform initial diagnostics and undertake the treatment necessary to stabilize the vital functions of patients who require medical assistance as a result of health- or life-threatening accident or illness. The treatment within hospital emergency departments should contribute to the reduction in mortality rate, the number and scope of disabilities caused by injuries, the number of deaths to be avoided, as well as the extent of pain and suffering resulting from accidents and sudden illnesses [11]. HEDs provide medical assistance to all patients in a state of emergency—both transported in ambulances and self-reporting, regardless of their age, place of residence, financial or social status.

In the event of a risk of loss of life or health, patients should be transferred to the hospital emergency department closest to the scene of the incident. Therefore, the problem of the distribution and access to emergency departments becomes of key importance. In this context, an important question seems to be whether the changes in the number and distribution of emergency departments, that take place after the implementation of the EMS in individual regions in Poland, result in an improvement in the level of spatial accessibility of the departments. This issue has not been widely addressed in the literature so far, although the results of such research could be a valuable hint for other countries with a similar level of development, reforming health and rescue systems for residents.

The aim of the paper is to explain the spatial accessibility of hospital emergency departments and changes in its level as a result of organizational and spatial transformations of the emergency department network in Poland. On the basis of the research results, it is possible to indicate new locations of emergency rooms, the launch of which would improve the level of spatial accessibility of emergency medical services. The research was conducted within two time series, comparing the changes in the distribution of HEDs in 2011 and 2021 and the resulting consequences for the level of spatial accessibility of these medical services. In the research procedure, GIS techniques were used to measure the distances between emergency departments and places of residence. Although the study was conducted on a local scale (approximately 2500 communes in Poland), the results were presented on a regional level.

## 2. Literature Review

### 2.1. Spatial Analysis of Emergency Department Accessibility

The emergency medical services is a field where spatial analysis is broadly used.

The crux of the emergency medical system is to save lives by providing a quick response to emergencies, thus minimizing the waiting time of the patient for professional emergency medical assistance, provided both in the pre-hospital stage (ambulance care) and as part of the hospital emergency care (i.e., in emergency departments). Shortening the time of ambulance response, patient transfer and rescue operation has clinical implications for many conditions. Yet increasing the chances of saving the patient depends in particular on the proximity to emergency units. Studies confirming this assumption have been conducted in numerous countries, e.g., Sweden [12], United Kingdom [13], Spain [14], the USA [15] and Portugal [16]. Their location should be well designed and result from the distribution and demographic structure of the population, terrain conditions, the socioeconomic situation and the existing and developing infrastructure. Geographic research on emergency medical services using quantitative methods to optimize location of the institutions focuses mainly on the aspect of spatial organization of the emergency medical system and its consequences [17,18].

In the case of pre-hospital emergency medical services, spatial analyses are related to the issues of access and effectiveness, which are closely connected to the distance expressed in patients’ waiting time for the ambulances to reach the places where accidents and other emergencies occur. Due to the possibility of relocating mobile emergency medical units, most of the works concern the determination of the optimal number and distribution of ambulance stations and the emergency ambulances themselves [19,20,21,22,23,24].

On the other hand, spatial studies of emergency medical services rendered at hospitals focus mainly on spatial, organizational and social aspects of the access to emergency departments. The relationships between the level of their spatial accessibility measured in travel time units and the level of availability as perceived by the local community are analyzed [25]. The authors state that time distance has an impact on the perceived level of availability, and it depends on the individual feelings of potential patients whether, when and where they will use the emergency department services.

Another important issue addressed in the context of distance to health care facilities is the changing demand for health care [26,27,28]. Vaz, Ramos and Santana [16] describe this phenomenon as “the distance decay effect”, resulting in decreasing demand for health care as distance to health care institutions increases. Brabyn and Beere [29] use GIS tools to calculate the closest emergency department in terms of travel time. They observe that after the hospital network reform in New Zealand, there are significant spatial inequalities in the accessibility of hospital emergency services, and the number of people living more than 60 min from the emergency department in some areas has increased dramatically. Spatial differentiation in the access to emergency departments is noticed by Carr et al. [30] and Hashtarkhani et al. [31]. The authors note a particularly unfavorable situation in rural areas and point at the need of further studies examining the relationship between the accessibility to emergency medical services in the context of mortality rate and medical results of patients in emergency departments.

The access to emergency medical services, taking into account both the ambulance arrival time to the scene and the time necessary to transport the patient to an emergency department, was tested by Jones and Bentham [4]. Using regression models to analyze data concerning rescue services for road accidents, they determined that an increased probability of death occurred when the accident participant was an elderly person (according to the model, over 60 years old), a pedestrian, or when it involved a larger number of cars or in the case of an accident on the road with a high permissible speed limit. Surprisingly, the study did not prove any relationship between the emergency action duration and the loss of life or health as a result of a road accident. The authors emphasized, however, that this could be the result of a limited research area, insufficient number of interventions analyzed, and difficulties in obtaining complete and reliable data.

The functioning of emergency departments has also been a hotspot of the current research concerning health care systems during COVID-19 pandemic and much new cognition about it has been yielded in the past two years. The increasing body of literature revealed that infrastructure problems, lack of access to medical essentials, and failures to deliver appropriate technological support were the key issues faced by health care organizations during this period. Recommendations to improve emergency department efficacy were made by numerous authors [32,33], while others focused on investigating changes in the numbers of visits to emergency departments during the pandemic and the reasons and results of these changes [34,35].

### 2.2. The Access to Health Services—Theoretical Background

Access to services has long been the subject of interest in many geographic, sociological and economic studies. Despite this, no exhaustive and unequivocal definition of the term has been developed so far, and most authors point out that the definitions available are vague or contradicting [36,37].

Domański [38] believes that the term access should be understood as “the possibility of taking advantage of opportunities created by economic, social, cultural and political objects and institutions”. The author notes that access is influenced by many factors, among which he distinguishes distance as a determinant of spatial accessibility. Similarly, Taylor [37], defines it as “opportunities or possibilities allowing the use of various types of activities, functions, some of which may be classified as services”.

When describing the concept of access, many authors consider, apart from the relations resulting from the location in space, the road network and the possibilities of transport as factors significantly determining the level of access [39,40,41].

A significant amount of attention has been paid to health services in spatial studies concerning accessibility. It can be stated that access to health services is equal to the possibility of obtaining advice or a medical procedure in a health care facility appropriate for the type of illness, with a minimum waiting time and minimum costs incurred. Therefore, the access to health services depends on the effective use of health care resources and their rational distribution, taking into account the population density, health condition of the population and the existing communication infrastructure and means of transport.

Most authors emphasize that access to health care is a complex concept that should be analyzed in several aspects (dimensions), among which the most important from the point of view of geographical research are spatial dimension (spatial accessibility) and the socio-organizational dimension (availability of services) [42,43,44,45,46]. Accessibility is identified as the relationship between the distribution of health care infrastructure and human resources and the distribution of the population reporting health needs. In turn, the availability of services is defined as the relationship between the sizes and types of services and the sizes and types of consumer needs, or sometimes as the adequate supply of medical services.

As defined above, the spatial accessibility depends on patients’ distance to the treatment site. Therefore, it is associated with transportation that requires devoting a certain time and incurring certain costs. Thus, spatial accessibility can be identified as the physical, time and economic distance to the place where medical services are rendered [42,44]. However, reaching a healthcare facility is not equal to getting medical help. There are a number of additional factors that determine the possibility of using health services. The utilization of medical services is often limited by such barriers as: organizational, financial, and, finally, personal [46].

In the case of hospital emergency departments responsible for providing medical services to every person in a situation of sudden health emergency, the organizational and socioeconomic dimensions of access seem to be less important than distance in determining spatial accessibility. On the one hand, there is a legal obligation to provide medical assistance to all patients admitted to emergency departments (which in principle translates into the lack of organizational and institutional barriers), and on the other hand, in the case of sudden and serious health threats, the timing of emergency assistance is of key importance. According to the so called “golden hour” principle applied in emergency medicine (the first hour after the onset of out-of-hospital traumatic injury), after the first 60 min of emergency, the patient’s chances of survival dramatically decrease. Therefore, one of the determinants of the effectiveness of emergency medical services is providing access to specialized hospital services to people in a state of sudden health emergency within no more than one hour [47]. Paying additional attention to the notion conceptualized in the literature, that the distribution of the population is the basic factor determining the spatial arrangement of the demand for health care [18,44,48,49], it can be concluded that when studying access to hospital emergency departments, attention should be focused primarily on spatial relations resulting from the location of emergency departments and the places of residence of potential patients.

## 3. Materials and Methods

The empirical data on the network of hospital emergency departments was obtained from two sources: (1) regional plans for the operation of the EMS, and (2) information on contracts concluded by individual Regional Departments of the National Health Fund. The preparation of the regional plans for the operation of the EMS in Poland is the task of voivodes (representatives of government administration in regions). They are the basic documents defining the organization and functioning of the system in the area of each region, and include, inter alia, information on the number, distribution and method of coordinating the activities of system units. The action plans of the EMS system served as a source of addresses of individual hospitals where HEDs operated and places where the emergency departments were planned to be launched. In turn, the National Health Fund (NHF), as a medical service payer, finances medical emergency services. The contract for the provision of services through the NHF is a prerequisite for including the hospital emergency department among the units of the EMS system. Data on concluded and implemented contracts for the provision of healthcare services in the field of hospital emergency department were collected from the NHF guide, which allowed for the verification of the HEDs actually functioning within the system. The remaining data necessary to analyse the spatial accessibility of hospital emergency departments were obtained using techniques related to geographic information systems (GIS) using the ArgGIS program. The usefulness of GIS tools in this type of research is emphasized by various authors [25,29,50,51,52,53,54].

The research procedure started with measuring the distance between each commune and the nearest hospital emergency department. Due to the nationwide scope of the research, measurements were made using the Euclidean metric (more precisely: orthodrome), although the authors were aware that the results of such measurements are almost always understated. Measurement in a straight line does not take into account the terrain conditions resulting from the route of the road network and the difference in altitude [55]. However, as the research of, e.g., Jones et al. [56] proves, the use of the Euclidean distance in spatial-medical research, despite the lower precision of the obtained measurements in relation to the so-called urban metric (distance measured along streets) is acceptable and does not distort the final results of the analyses.

When determining the distance, it was necessary to define points representing the individual commune. In the case of urban communes (municipalities), it was the mean centre of the city. In rural communes it was the population mean centre of the commune, while in urban-rural communes, it was the mean centre of the city being the seat of the commune.

In the next step, the data were aggregated at the regional level and the weighted average distances of places of residence to emergency departments were calculated according to the formula:

(1) $${\bar{d}}_{\mathrm{HED}}=\frac{\sum_{i=1}^{n}d_{i}P_{i}}{\sum_{i=1}^{n}P_{i}},$$

where *d_i_* is the distance to the HED from a given commune, and *P_i_* is the number of people living in particular communes. In addition, each of the communes was included in one of the five zones of distance (remoteness) from the HED. The zones were separated on the basis of previous measurements, setting limit values every 10 km. Then, the number of people living in individual zones was summed up, both at the country level and in individual regions, and the results were presented as a percentage.

**Figure 1:**
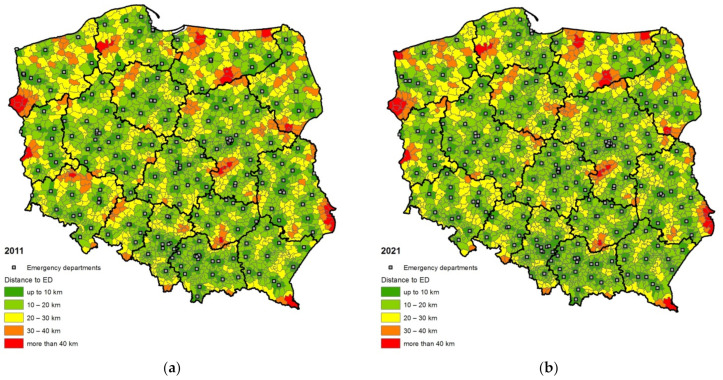
Spatial accessibility of hospital emergency departments in the Polish communes: (**a**) in 2011; (**b**) in 2021.

The study was conducted in two time series covering 2011 and 2021 (Figure 1). On this basis, the changes in the level of access resulting from organizational changes within the hospital emergency system were compared.

## 4. Results

### 4.1. The Hospital Emergency Department Network in Poland

There were assumptions regarding the organization of the emergency medical service system in Poland forecasting the creation of approximately 270 hospital emergency departments, each of them covering the population from 100,000 to 300,000 with its catchment area [9,57]. Despite the relevant provisions in the regional action plans for the system, many Polish hospitals failed to meet the organizational criteria imposed by the legislator to launch the HED.

In individual regions, the organizers of the EMS system made different assumptions regarding the number and distribution of the emergency departments. The general rule was to launch HEDs in the largest population centers. In this way, emergency departments were established in administrative capitals of the regions and in all the biggest cities in the country.

Among the cities with over 100,000 residents without HED were Gliwice, Bytom, Ruda Śląska and Tychy (in 2011 also the city of Zabrze). These were the cities of the Upper Silesian urban area—the largest urban area in Poland and one of the largest in the European Union. The services of hospital emergency departments in the conurbation area were secured in ten other cities. The lack of hospitals with emergency departments was also noticeable in areas adjacent to large cities (including the surroundings of Rzeszów, Olsztyn, Szczecin, Bydgoszcz, Toruń). Patients from these areas were sent to HEDs operating in the central cities.

**Figure 2:**
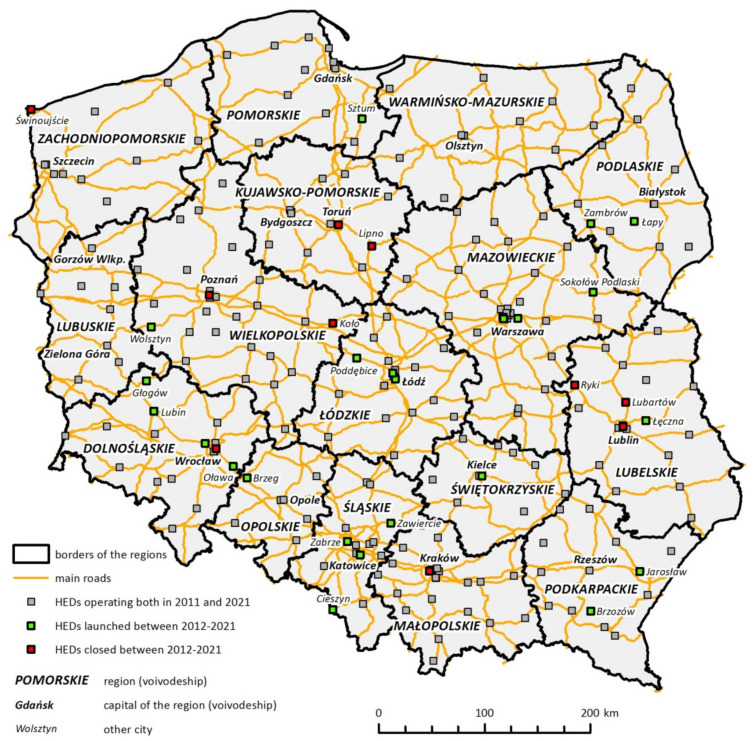
Spatial distribution of hospital emergency departments in Poland.

In some regions efforts were made to ensure that HEDs were located in almost every district (poviat) (e.g., in the Małopolskie region), in others the focus was on launching emergency departments in places of the greatest population concentration (e.g., the Kujawsko-Pomorskie and Śląskie regions). When planning the location of departments, it was also important to take into consideration the structure of the national road network and highways (e.g., the Lubuskie, Dolnośląskie, Wielkopolskie regions) (Figure 2).

In 2011, out of 256 planned emergency departments, 229 (almost 90%) were in operation. The plans for the organization of HEDs were fully implemented in several regions: Kujawsko-Pomorskie, Lubelskie, Lubuskie, Łódzkie, Małopolskie and Opolskie. Slight differences in relation to the assumptions occurred in the Pomorskie, Podlaskie, Warmińsko-Mazurskie, Wielkopolskie and Zachodniopomorskie regions (each region did not activate one of the planned HEDs). The lowest number of emergency departments completed was observed in the Śląskie region, where by 2011 only 10 out of 23 planned departments (43%) were established. A relatively low degree of implementation of the adopted assumptions was also recorded in the Dolnośląskie (81%) and Świętokrzyskie (82%) regions (Table 1).

In the following years, the hospital emergency department network was transformed. Both the number of HEDs planned by voivodes and the number of departments under operation changed. In some regions, the emergency medical system planners amended the assumptions regarding the number and distribution of emergency departments in the action plans. While shaping the network of emergency departments, on the one hand, efforts were made to maximize spatial accessibility, and on the other hand, the economic efficiency of providing emergency and medical services was taken into account.

As a result of the ongoing organizational and spatial transformation up to 2021, a network of hospital emergency departments included 241 out of the planned 267 venues. The degree of implementation of the assumptions stipulated in the plan exceeded 90%, and this value did not differ significantly from that from 10 years ago. Compared to 2011, the number of regions where all emergency departments were launched (the Dolnośląskie, Lubuskie, Małopolskie, Opolskie and Wielkopolskie regions) decreased. An almost complete network of HEDs (only one department less than planned) operated in the Podkarpackie, Pomorskie, Świętokrzyskie and Warmińsko-Mazurskie regions. As in 2011, the lowest effectiveness in launching the planned number of emergency departments could be observed in the Śląskie region, yet the degree of implementation of the HED network increased in this voivodeship from 43% to 67%. Some difficulties could also be noted in the Lubelskie, Łódzkie and Mazowieckie regions, where three emergency departments remained not launched. In the remaining regions, two departments were missing to complete the plan for the construction of the HED network (Table 1).

### 4.2. Spatial Accessibility of Hospital Emergency Departments

The analysis of spatial accessibility of hospital emergency departments in 2011 showed that over half of the Polish population (52.4%) lived within 10 km from the nearest emergency department. In municipalities included in the further zones, 28.2% of the total population of the country lived 10–20 km from HEDs, 15.2% had to travel the distance of 20–30 km, 3.5% had to cover the distance of 30–40 km, and only 0.7% of the population had to travel the distance of more than 40 km to the closest HED (Table 2). The weighted average distance between emergency departments and places of residence, calculated on the basis of assumptions made, was 11 km (Table 3). This value indicated a relatively high spatial accessibility of hospital emergency departments in Poland, especially in a situation where one of the main factors taken into account in the process of shaping the network of emergency departments was economic efficiency leading to the concentration of services and the construction of health facilities mainly in the largest cities.

The conducted analysis showed quite significant regional differentiation in terms of spatial accessibility of HEDs, measured by the distance separating the potential patients from the nearest hospital with an emergency department. The shortest distances in 2011 were noted in the regions of Łódzkie (${\bar{d}}_{\mathrm{HED}}$ = 8 km) and Małopolskie (${\bar{d}}_{\mathrm{HED}}$ = 8.5 km), where over 60% of the population lived in the communes located within 10 km away from the HED, and less than 1% of the population had to travel the distance greater than 30 km to reach it. The weighted average distance between emergency departments and places of residence, apart from the above-mentioned regions, did not exceed 10 km in the Mazowieckie (9.2 km) and Pomorskie (10 km) regions. The lowest spatial accessibility of emergency departments was observed in the regions of Opolskie (15.4 km), Warmińsko-Mazurskie (15.2 km) and Zachodniopomorskie (15.2 km). The first of the distinguished zones of remoteness from the HED (up to 10 km) was inhabited by only every third resident of Opolskie (33.5%) and slightly more than 40% of Warmińsko-Mazurskie. In turn, as many as 5.7% of the population in the Warmińsko-Mazurskie and over 2% in the Lubuskie, Lubelskie and Zachodniopomorskie regions lived further than 40 km from the HED (Table 2 and Table 3).

The repeated research procedure regarding 2021 proved that along with the transformation of the network of hospital emergency departments in Poland, the level of spatial accessibility of medical services in Poland changed. The progressing spatial reorganization of emergency departments resulted in an increase in the percentage of the population living in the two closest zones away from emergency departments—in the up to 10 km zone by more than 2 percentage points, and in the 10–20 km zone by 0.4 percentage points. On the other hand, the share of the population living in the areas farthest from the functioning HEDs decreased—in the 20–30 km zone there was a decrease by 2 percentage points, while in the 30–40 km zone by 0.7 percentage point (Table 2). The study showed that the weighted average distance between the places of residence and the nearest hospital emergency department decreased by 0.6 km on the national scale and in 2021 amounted to 10.4 km (Table 3).

The analysis of regional differentiation in terms of spatial accessibility measured by the average weighted distance to the nearest emergency department showed that in 2021 the lowest values of the indicator were characteristic of the regions: Łódzkie (7.9 km), Mazowieckie (8.7 km), Małopolskie (8.9 km), Śląskie (9.3 km) and Pomorskie (9.3 km). In turn, the greatest average distances to hospital emergency departments were identified in the Zachodniopomorskie (16.6 km), Warmińsko-Mazurskie (15.1 km) and Opolskie (13 km) regions.

Comparing the results of the analyses carried out on the data from 2011 and 2021, it can be observed that in most regions changes in the distribution of emergency departments led to a decrease in the average distance between the places of residence and emergency departments (Table 3). The greatest reduction in weighted average distance was observed in the Dolnośląskie and Opolskie regions, where, thanks to the opening of additional emergency departments, the ${\bar{d}}_{\mathrm{HED}}$ indicator decreased by over 2 km. To a lesser extent, due to the changes in the spatial organization of hospital emergency departments, the average distance to this type of facilities was shortened in the following regions: Śląskie (almost 2 km), Podlaskie (1.4 km), Podkarpackie (1.3 km) and Pomorskie (approx. 0.7 km). In turn, in Zachodniopomorskie and Kujawsko-Pomorskie the weighted average distance to the nearest emergency department increased by 1.4 km and 1 km, respectively. In other regions no significant changes in the level of spatial accessibility of hospital emergency departments were recorded in 2021 compared to 2011 (changes ranged within +/− 0.5 km).

In the analysed decade, particularly favorable changes took place in the Śląskie and Dolnośląskie regions, where the share of population living within 10 km from the HED increased by 10.4 and 7.4 percentage points, respectively. In the Śląskie region, it happened due to the launch of emergency departments in Cieszyn, Zawiercie and Zabrze, and further improvement is expected due to the implementation of the next seven HEDs to be launched (including Racibórz, Jastrzębie-Zdrój, Żywiec and Tychy). In turn, in the Dolnośląskie region, the improvement in the accessibility of emergency services resulted from fully implemented plans for the network of emergency departments, which in recent years were expanded by the departments in Lubin, Głogów and Oława. A different situation was observed in the Zachodniopomorskie and Kujawsko-Pomorskie regions, where the liquidation of HEDs decreased the percentage of the population dwelling the in first zone of remoteness by almost 3 percentage points.

The analysis of the spatial accessibility of HEDs in Poland proved that a decreasing share of the population lives in further zones of remoteness. There are exceptions to this rule in two regions—Warmińsko-Mazurskie and Zachodniopomorskie. In these regions, the network of HEDs is not fully adjusted to the population distribution. In Warmińsko-Mazurskie, the situation will be improved by launching the planned HED in Szczytno. It seems that an additional location in Ostróda and Braniewo would be worth considering. In the Zachodniopomorskie region decision makers should consider locating new HEDs in Świnoujście, Kołobrzeg and Myślibórz (locations not included in the plan).

Among other locations where the launch of the HED would significantly reduce the percentage of people living in the farthest zones of remoteness, and thus contribute to the reduction in the average distance to the emergency department, one should mention Krosno Odrzańskie in the Lubuskie region (not included in the plan), Grójec in the Mazowieckie region (planned to be launched), Hrubieszów in the Lubelskie region (planned) and Busko-Zdrój in the Świętokrzyskie region (planned).

## 5. Discussion

The presented changes in the number and distribution of emergency departments indicate that despite the dozen or so years of operation of the EMS system in Poland, the network of emergency departments has not yet reached its final state. Generally, it is difficult to identify any visible regularities in the degree of plan implementation for the organization of the network of emergency departments in individual regions. Most often, the failure to open the HED was explained by financial problems and staff shortages. It stems from the fact that there are a number of structural, space and organizational requirements necessary for the functioning of the HED. These criteria indicate the minimum number of staff of emergency departments and stipulate, inter alia, that these departments are organized in hospitals with anesthesiology and intensive care units, general surgery with trauma and internal diseases, as well as 24-h laboratory diagnostics and diagnostic imaging. The hospital should also have access to a helipad for medical helicopters [10].

Initially, a big problem was to adapt the emergency departments to the technical requirements (proper location of the emergency department in the hospital structure, appropriate usable floor area, entrance for ambulances, proper patient flow inside the HED). Currently, a major barrier to the functioning of HEDs is the provision of minimum human resources, including primarily those relating to doctors in the system [58,59,60,61]. At the same time, it seems that the number of operating HEDs was largely affected by the determination and effectiveness of the directors of individual hospitals as well as the support for the idea by decision makers in each of the region [57].

Despite the above-mentioned problems, the transformation of the spatial organization of hospital emergency department network in 2011–2021 affected the level of accessibility of these facilities, both in the scale of the entire country and in the system of individual regions.

The applied research procedure proved that the introduced changes resulted in the overall improvement of the spatial accessibility of the hospital part of the medical emergency system, reducing the weighted average distance between the places of residence and the nearest HED (on the national scale by 0.6 km, and in some regions even by a few km). These results allow for a positive assessment of the changes in the distribution of emergency departments, especially in the context of the view emphasized in the literature that the distribution of the population is the basic factor determining the spatial arrangement of the demand for health services [18,44,48,49]. Numerous studies prove that distance has a negative influence on HED utilization [16,51,62,63], and decreased emergency department access can be reflected in a substantial increase in the mortality rate [7,64,65,66].

The relatively small distances between the places of residence and HEDs were the result of their presence in large cities, where a significant part of the population of individual regions lives. It seems that in large cities it was easier to meet the requirements of both qualified staff and professional technical and infrastructure facilities, necessary to start the HED. A much greater problem in the largest cities is the availability of HEDs resulting from their excessive use and the excessive transfer time resulting from transport congestion [67,68]. On the other hand, in small towns and in rural areas, a greater problem is the economic efficiency of emergency departments and the shortage of physicians of EMS and specialist equipment. Yet as numerous studies emphasize, the departments in these locations are important safety nets for members of the local community [69]. Their functioning is also of key importance in case of road accident victims [4].

In the context of EMS for accident victims and patients with sudden illnesses in Poland, other hospital units cooperating with the system should also be mentioned. These include trauma centers, departments in specialized hospitals, such as invasive cardiology, neurosurgery, thoracic surgery, maxillary surgery, toxicology and stroke treatment. Some authors [70] stress that getting to the right specialist center as soon as possible, even if it is not the nearest HED, increases the patient’s chances of survival. However, due to the uniqueness of services provided in these centers, they should not be included in the analysis of the availability of hospital emergency departments [71]. Moreover, trauma centers and a large number of specialist departments operate in the same hospitals where HEDs are located, therefore even including them in the adopted research procedure would not change the obtained results.

In hospitals where, for various reasons, it was not possible to organize an emergency department, alternative services are rendered in the admission room [10]. However, as emphasized by Gaca and Witkowski [57], the scope of these services is different and is not subject to the provisions of the Act on the EMS.

The presence of an emergency department in a given area that meets the criteria imposed by the legislator significantly improves the quality and availability of medical services offered, and thus increases the local residents’ sense of security, by being aware of the existence of an institution that is constantly in the mode of readiness [11,57].

## 6. Conclusions

The Polish system of emergency medical services, introduced by the Act on the Emergency Medical Services of 2006, was characterized by many changes in the number and arrangement of facilities with hospital emergency departments in the studied period. In the light of the research results obtained, it was found that despite the still unfinished plans to build a network of HEDs in Poland, changes in the number and distribution of departments are aimed at improving the spatial accessibility of these medical services. It is manifested by the reduction in the weighted average distance separating emergency departments from the places of residence. A further increase in accessibility should be expected after launching the planned departments, especially in peripheral cities in several regions.

In order to ensure high quality and access to hospital emergency medical services, after bringing the network of HEDs to the target state, further research should focus on the availability of services, with particular emphasis on the degree of services’ utilization in the departments.

Although the level of spatial accessibility of emergency departments increased on the country level and in most regions in the analyzed period, there are also regions where reverse trends were identified. This is due to department closures or failure to complete the planned network. The situation is particularly unfavorable in the Zachodniopomorskie region, where the existing HED network proved to be insufficient, and the loss of HED in the hospital in Świnoujście further reduced the spatial accessibility of the analyzed medical services.

Although the study of spatial accessibility of hospital emergency departments was limited only to issues related to the physical distance separating the HEDs from the places of residence, it should be emphasized that in the case of sudden health threats this distance significantly determines the overall waiting time for emergency services. Therefore, the results of the presented research may be the basis for the spatial evaluation of the emergency department network. Minimizing the average distances to HEDs may be a goal function when planning the launch of subsequent departments. Such an approach will make it possible to improve the spatial accessibility of the hospital base of emergency medical services, thus contributing to better protection of the health needs of residents.

## Figures and Tables

**Table 1:** The network of hospital emergency departments in Polish regions.

Region	No. of HEDs in 2011			No. of HEDs in 2021		
	Functioning	Planned	Level of Completion	Functioning	Planned	Level of Completion
Dolnośląskie	13	16	81%	16	16	100%
Kujawsko-Pomorskie	12	12	100%	10	12	83%
Lubelskie	19	19	100%	17	20	85%
Lubuskie	8	8	100%	8	8	100%
Łódzkie	17	17	100%	20	23	87%
Małopolskie	22	22	100%	21	21	100%
Mazowieckie	30	32	94%	32	35	91%
Opolskie	6	6	100%	7	7	100%
Podkarpackie	12	14	86%	14	15	93%
Podlaskie	11	12	92%	13	15	87%
Pomorskie	12	13	92%	13	14	93%
Śląskie	10	23	43%	14	21	67%
Świętokrzyskie	9	11	82%	10	11	91%
Warmińsko-Mazurskie	11	12	92%	11	12	92%
Wielkopolskie	26	27	96%	25	27	93%
Zachodniopomorskie	11	12	100%	10	10	100%
**POLAND**	**229**	**256**	**89%**	**241**	**267**	**90%**

Source: own study based on 16 regional action plans of the Emergency Medical Services and the information on National Health Fund contracts.

**Table 2:** Spatial accessibility of the hospital emergency departments in Poland.

Region	Share of Population Living within a Given Distance from the Nearest HED									
	2011					2021				
	Up to 10 km	10–20 km	20–30 km	30–40 km	>40 km	up to 10 km	10–20 km	20–30 km	30–40 km	>40 km
Dolnośląskie	45.6	26.0	23.6	4.5	0.2	53.0	29.8	16.5	0.6	0.0
Kujawsko-Pomorskie	53.7	25.9	17.8	2.6	0.0	50.9	24.8	20.0	4.3	0.0
Lubelskie	51.9	24.4	18.1	3.3	2.2	51.4	23.2	20.3	3.1	2.1
Lubuskie	47.6	22.9	23.6	3.5	2.4	48.0	24.0	22.3	3.4	2.3
Łódzkie	60.7	26.8	11.6	0.8	0.0	60.9	28.1	10.1	0.9	0.0
Małopolskie	60.8	33.5	5.1	0.5	0.0	60.4	33.8	5.3	0.5	0.0
Mazowieckie	58.9	26.4	10.0	4.3	0.3	60.5	27.8	8.0	3.5	0.3
Opolskie	33.5	32.6	23.5	10.5	0.0	39.1	35.0	24.1	1.7	0.0
Podkarpackie	45.1	39.1	15.6	0.1	0.1	50.2	40.8	8.8	0.1	0.1
Podlaskie	52.8	22.1	17.1	6.3	1.8	58.7	21.0	13.4	5.2	1.7
Pomorskie	57.6	22.0	15.4	4.0	1.1	58.2	26.0	13.2	1.6	1.0
Śląskie	50.2	35.7	13.3	0.7	0.0	60.7	30.0	9.0	0.3	0.0
Świętokrzyskie	50.2	30.7	12.4	6.2	0.4	49.6	31.4	13.3	5.3	0.4
Warmińsko-Mazurskie	42.8	17.5	26.3	7.8	5.7	42.0	18.8	26.0	7.6	5.7
Wielkopolskie	52.5	31.3	14.2	2.0	0.0	51.7	32.0	13.5	2.9	0.0
Zachodniopomorskie	46.1	16.7	23.6	11.5	2.1	43.4	19.3	20.5	12.1	4.8
**POLAND**	**52.4**	**28.2**	**15.2**	**3.5**	**0.7**	**54.7**	**28.6**	**13.2**	**2.7**	**0.8**

Source: own calculation on the basis of data acquired from Statistics Poland.

**Table 3:** Weighted average distances to hospital emergency departments.

Region	2011	2021
Dolnośląskie	13.2	10.7
Kujawsko-Pomorskie	10.6	11.6
Lubelskie	11.9	12.4
Lubuskie	12.6	12.5
Łódzkie	8.0	7.9
Małopolskie	8.5	8.9
Mazowieckie	9.2	8.7
Opolskie	15.4	13
Podkarpackie	11.4	10.1
Podlaskie	11.6	10.2
Pomorskie	10.0	9.3
Śląskie	11.1	9.3
Świętokrzyskie	11.1	11.1
Warmińsko-Mazurskie	15.2	15.1
Wielkopolskie	10.2	10.7
Zachodniopomorskie	15.2	16.6
**POLAND**	**11.0**	**10.4**

Source: own calculation on the basis of data acquired from Statistics Poland.

## Data Availability

The data supporting reported results can be found at websites of Statistics Poland stat.gov.pl (accessed on 1 December 2021) and National Health Fund nfz.gov.pl (accessed on 1 December 2021).
